# Quasi-integrability and nonlinear resonances in cold atoms under modulation

**DOI:** 10.1098/rsos.231503

**Published:** 2024-04-10

**Authors:** Rahul Gupta, Manan Jain, Sudhir R. Jain

**Affiliations:** ^1^ Department of Physics, Indian Institute of Technology, Mumbai 400076, India; ^2^ School of Physics and Astronomy, University of Birmingham, Birmingham B15 2TT, UK; ^3^ UM-DAE Centre for Excellence in Basic Sciences, University of Mumbai, Vidyanagari Campus, Mumbai 400098, India

**Keywords:** ultracold atoms, quasi-integrability, semiclassical methods, quantum chaos, dynamical localization

## Abstract

Quantum dynamics of a collection of atoms subjected to phase modulation has been carefully revisited. We present an exact analysis of the evolution of a two-level system (represented by a spinor) under the action of a time-dependent matrix Hamiltonian. The dynamics is shown to evolve on two coupled potential energy surfaces (PESs): one of them is binding, while the other one is scattering type. The dynamics is shown to be quasi-integrable with nonlinear resonances. The bounded dynamics with intermittent scattering at random moments presents a scenario reminiscent of Anderson and dynamical localization. We believe that a careful analytical investigation of a multi-component system that is classically non-integrable is relevant to many other fields, including quantum computation with multi-qubit systems.

## 1. Introduction

Evolution in the fields of ultracold atoms and quantum physics in the past few decades has led to the recognition of these fields as a huge well-acclaimed arena for the exploration of popular subjects like quantum chaos [1], Feshbach resonances [2–12], ultracold atomic mixtures [13–18], atom interferometry [19–33], atomic clocks [34–44], quantum diffraction [45,46] and quantum thermodynamics [47–50]. This is due to the rich internal structures, longer de Broglie wavelengths and tunable long-range interactions of ultracold atoms. Furthermore, the research in the regime of lower temperatures has also been extended to these molecules [51,52]. Apart from these recent developments, there has been a sustained effort to realize parallels between atomic and condensed matter physics [53]. One of the ideas pursued with great interest is the localization of states in disordered systems, pioneered by Anderson [54]. Due to a common-sense analogy between disorder and chaos, a connection between the localization of wavefunctions of classically chaotic systems and the disordered lattices of infinite [55] and finite extent [56] was brought out. Even in matter waves, the phenomenon of localization has been experimentally demonstrated [57].

Many years ago, an experiment carried out by the group led by Raizen [1] demonstrated the dynamical analogue of Anderson localization in a system of cold atoms. In this experiment, approximately 100 000 ^23^Na atoms were trapped in a spherical volume of radius 300 µm at a temperature of 17 µK. At the end of the preparation step, the cooling lasers were turned off and a modulated standing light field was switched on for 10 µs. The Hamiltonian describing the interaction of a sodium atom of mass *m* at position *x* and momentum *p* with the light field is given by [58]


(1.1) $$H_{0}=H_{el}+\frac{p^{2}}{2m}+eF\cos\{k_{L}[x-\Delta L\sin\omega t]\}\cos\omega_{L}t.$$

Here, $H_{el}$ denotes the interaction of valence electrons with an atom. The last term denotes the electric dipole interaction of the electromagnetic field with an electron. The laser frequency and wavenumber are denoted by $\omega_{L}$ and $k_{L}$ , respectively, and ω is the modulation frequency. Standing waves are generated by directing two counter-propagating laser beams into the trap and the modulation is achieved by passing one beam through an electro-optical phase modulator. The beam is made to strike a mirror in a cavity of length ΔL that is moving with the modulation frequency, ω . The laser frequency was chosen close to the D_2_ line of sodium. The electronic Hamiltonian can be reduced to a two-level system written on the basis of a ground state $|g\rangle =(0,1{)}^{T}$ and an excited state, $|e\rangle =(1,0{)}^{T}$ , such that a general state with complex amplitudes $\psi^{+}$ and $\psi^{-}$ in the respective two levels can be written as $\psi =\psi^{+}|e\rangle +\psi^{-}|g\rangle$ . Taking the energy average of the two states as zero energy, the matrix elements of $H_{el}$ and eF together give


(1.2) $$H_{el}=\frac{\hbar \omega_{0}}{2}\sigma_{z}\;\;;\;\;eF=\hbar \Omega \sigma_{x}\;\;⟹\;\;H_{el}+eF=\left(\begin{array}{cc} \hbar \omega_{0}/2 & \hbar \Omega \\ \hbar \Omega & -\hbar \omega_{0}/2 \end{array}\right),$$

where the transition frequency between these two levels is denoted by $\omega_{0}$ , Ω denotes the Rabi frequency coupling the two states by electric dipole interaction and $\sigma^{\prime }s$ are the Pauli matrices. Thus, *H*_0_ may be written as


(1.3) $$H_{0}=\frac{p^{2}}{2m}I+\frac{\hbar \omega_{0}}{2}\sigma_{z}+\hbar \Omega \cos\{k_{L}[x-\Delta L\sin\omega t]\}\cos(\omega_{L}t)\sigma_{x},$$

where I denotes an identity matrix.

After we present the general Hamiltonian below, in §2, we present the Hamiltonian under rotating wave approximation (RWA). Within this approximation, the case of adiabatic perturbation for the two cases of small and large detuning is considered. In §3, the exact solution for this matrix Hamiltonian is given. The method transforms the dynamics under the matrix Hamiltonian to dynamics on potential energy surfaces (PESs). Classical dynamics reveals the presence of nonlinear resonances in §4. The classical system obeys the Kolmogorov–Arnold–Moser (KAM) theorem [59] and hence is quasi-integrable [60]. In a related context of the quantum Rabi model, a discussion on integrability [61] and symmetries [62] has been presented relatively recently.

Special solutions are discussed as they have been used to analyse experiments carried out by different groups. For each case discussed at the quantum mechanical level, we also present classical phase space pictures and show that this atomic system presents a very interesting and deep instance of the association of quasi-integrability and dynamical localization. The phase space pictures exhibit certain misleading features in the approximated Hamiltonian, compared with the exact Hamiltonian obtained by systematic expansion in powers of ℏ .

### 1.1. General Hamiltonian

We now transform to a frame which is rotating with $\omega_{L}$ about the z -axis in a spin space,


(1.4) $$\psi_{rot}=\exp\left(i\omega_{L}\sigma_{z}t/2\right)\psi .$$

Substituting ψ in the Schrödinger equation, $i\hbar \partial \psi /\partial t=H_{0}\psi$ , we have the equation for the rotated wavefunction,


(1.5) $$H_{rot}=\frac{p^{2}}{2m}I+\frac{\hbar (\omega_{0}-\omega_{L})}{2}\sigma_{z}+\hbar \Omega \cos\{k_{L}[x-\Delta L\sin\omega t]\}\times \;\times \cos(\omega_{L}t)e^{i\omega_{L}\sigma_{z}t/2}\sigma_{x}e^{-i\omega_{L}\sigma_{z}t/2}.$$

Using the standard identity, $e^{i\omega_{L}\sigma_{z}t/2}\sigma_{x}e^{-i\omega_{L}\sigma_{z}t/2}=\sigma_{x}\cos\omega_{L}t-\sigma_{y}\sin\omega_{L}t$ , we have the transformed Hamiltonian,


(1.6) $$H_{rot}=\frac{p^{2}}{2m}I+\frac{\hbar (\omega_{0}-\omega_{L})}{2}\sigma_{z}+\frac{\hbar \Omega }{2}\cos\{k_{L}[x-\Delta L\sin\omega t]\}\times \;\times [\sigma_{x}(1+\cos2\omega_{L}t)-\sigma_{y}\sin2\omega_{L}t].$$

This is the general Hamiltonian for the physical situation described above where there are terms oscillating with twice the $\omega_{L}$ .

## 2. Rotating wave approximation

The Schrödinger equation for $H_{rot}$ is usually solved under the RWA [58,63]. Here, the terms oscillating with frequency $2\omega_{L}$ are neglected. This leads to a simplified Hamiltonian,


(2.1) $$H_{rot}^{RWA}=\frac{p^{2}}{2m}I+\hbar \Omega_{eff}(\sigma_{z}\cos\alpha +\sigma_{x}\sin\alpha ),$$

where


(2.2) $$\Omega_{eff}=\frac{1}{2}[(\omega_{0}-\omega_{L}{)}^{2}+\Omega^{2}\cos^{2}\{k_{L}(x-\Delta L\sin\omega t)]\}{]}^{1/2},\;\tan\alpha =\frac{\Omega \cos[k_{L}(x-\Delta L\sin\omega t)]}{\omega_{0}-\omega_{L}}.$$

Let us rotate the state of this Hamiltonian further in the spin space by an angle (−α/2) about the *y*-axis, to obtain a new state, $\psi^{\prime }=\psi^{\prime +}|e\rangle +\psi^{\prime -}|g\rangle =\exp(i\alpha \sigma_{y}/2)\psi_{rot}$


(2.3) $$\psi^{\prime }=\left(\begin{array}{c} \cos(\alpha /2)e^{i\omega_{L}t/2}\psi^{+}+\sin(\alpha /2)e^{-i\omega_{L}t/2}\psi^{-}\\ -\sin(\alpha /2)e^{i\omega_{L}t/2}\psi^{+}+\cos(\alpha /2)e^{-i\omega_{L}t/2}\psi^{-} \end{array}\right),$$

in which the second term is diagonal. Consequently, the equation satisfied by $\psi^{\prime }$ is


(2.4) $$i\hbar \frac{\partial \psi^{\prime }}{\partial t}=-\frac{\hbar }{2}\frac{\partial \alpha }{\partial t}\sigma_{y}\psi^{\prime }+e^{i\alpha \sigma_{y}/2}H_{rot}^{RWA}e^{-i\alpha \sigma_{y}/2}\psi^{\prime }=H_{eff}^{RWA}\psi^{\prime }.$$

However, this will transform the kinetic term as [64]


(2.5) $$e^{i\alpha \sigma_{y}/2}p^{2}Ie^{-i\alpha \sigma_{y}/2}\psi^{\prime }={\left(pI-\hbar A\right)}^{2}\psi^{\prime }=\Pi^{2}\psi^{\prime },$$

(2.6) $$A=\frac{\sigma_{y}}{2}\frac{\partial \alpha }{\partial x}=\frac{-k_{L}\delta_{L}\Omega \sin[k_{L}(x-\Delta L\sin\omega t)]\sigma_{y}}{2\left({\delta_{L}}^{2}+\Omega^{2}\cos^{2}[k_{L}(x-\Delta L\sin\omega t)]\right)}.$$

where I is an identity matrix. Now, we can use the well-known identity


(2.7) $$e^{i\alpha (\hat{n}.\vec{\sigma })}\vec{\sigma }e^{-i\alpha (\hat{n}.\vec{\sigma })}=\vec{\sigma }\cos2\alpha +\hat{n}\times \vec{\sigma }\sin2\alpha +\hat{n}(\hat{n}.\vec{\sigma })(1-\cos2\alpha ).$$

While the ‘potential’ part of the Hamiltonian becomes diagonal with these unitary transformations, the kinetic term modifies to $(pI-\hbar A{)}^{2}$ . This has terms of order 1, ℏ and $\hbar^{2}$ ; thus, an asymptotic semiclassical expansion appears in a natural manner [65–67]. The asymptotic expansion parameter ℏ [64] is small compared with the relevant classical action.^1^ It is worth noting that this powerful method has been successfully used to obtain the ‘exact’ ground states for deuteron [68] and triton [69]. Moreover, we would like to recall that the semiclassical trace formula for oscillator potentials gives the exact level density, where one performs an asymptotic expansion of the energy-dependent Green function or propagator in powers of ℏ [70].

Furthermore, since A has non-zero diagonal matrix elements, there is a possibility of a geometric phase appearing in the state of the atoms as the system evolves. This is indeed due to the cavity modulation. Dimensionally, ℏA/e is a magnetic vector potential. $H_{eff}^{RWA}$ can be written as


(2.8) $$H_{eff}^{RWA}=\frac{\Pi^{2}}{2m}+\hbar \Omega_{eff}\sigma_{z}-\frac{\hbar }{2}\frac{\partial \alpha }{\partial t}\sigma_{y},$$

(2.9) $$\;=\left[\frac{p^{2}}{2m}+\frac{\hbar^{2}}{8m}{\left(\frac{\partial \alpha }{\partial x}\right)}^{2}\right]I+\hbar \Omega_{eff}\sigma_{z}+\left(-\frac{\hbar }{2}\frac{\partial \alpha }{\partial t}-\frac{\hbar }{2}\frac{\partial \alpha }{\partial x}\frac{p}{m}+\frac{i\hbar^{2}}{2m}\frac{\partial \alpha }{\partial x}\right)\sigma_{y}.$$

Except for terms of order O( $\hbar^{2}$ ), each of the terms can make a significant contribution. At this point, one of the possible simplifications occurs if α is slowly varying with time. This leads us to consider applying the adiabatic approximation, which we will discuss now.

### 2.1. Adiabatic variation

We may neglect the term $\hbar \sigma_{y}d\alpha /dt$ . Here, we invoke the classical correspondence of dx/dt and p /m by writing^2^


(2.10) $$\hbar \sigma_{y}\frac{d\alpha }{dt}\to \hbar \frac{\partial \alpha }{\partial x}\frac{p}{m}\sigma_{y}+\hbar \frac{\partial \alpha }{\partial t}\sigma_{y},$$

which is small for an adiabatic variation. The adiabatic Hamiltonian is


(2.11) $$H_{ad}^{RWA}=\frac{1}{2m}\left[p^{2}+\frac{\hbar^{2}}{8m}{\left(\frac{\partial \alpha }{\partial x}\right)}^{2}\right]I+\hbar \Omega_{eff}\sigma_{z}+\frac{i\hbar^{2}}{2m}\frac{\partial \alpha }{\partial x}\sigma_{y}.$$

It is important if the detuning is small or large. This is because


(2.12) $$\frac{\partial \alpha }{\partial x}=-\frac{k_{L}\frac{\delta_{L}}{\Omega }\sin[k_{L}(x-\Delta L\sin\omega t)]}{{\left(\frac{\delta_{L}}{\Omega }\right)}^{2}+\cos^{2}[k_{L}(x-\Delta L\sin\omega t)]};\;\frac{\partial \alpha }{\partial t}=\frac{\omega \frac{\delta_{L}}{\Omega }\sin[k_{L}(x-\Delta L\sin\omega t)]\cos\omega t}{{\left(\frac{\delta_{L}}{\Omega }\right)}^{2}+\cos^{2}[k_{L}(x-\Delta L\sin\omega t)]}.$$

So either for small or large detuning,


(2.13) $$\delta_{L}\ll \Omega \;or\;\delta_{L}\gg \Omega \;\Rightarrow \;\frac{\partial \alpha }{\partial t},\frac{\partial \alpha }{\partial x}\to 0.$$

#### 2.1.1. Small detuning

Here, $\omega_{0}∼\omega_{L}$ , thus tan⁡α→∞ or α∼π/2 . Considering equation (2.13) and keeping the terms up to O (ℏ) , the adiabatic Hamiltonian further simplifies to


(2.14) $$H_{ad,s}^{RWA}=\frac{p^{2}}{2m}I+\hbar \Omega_{eff}\sigma_{z}.$$

Using the smallness of detuning, we may expand it binomially to obtain


(2.15) $$H_{ad,s}^{RWA,\pm }=\frac{p^{2}}{2m}\pm \frac{\hbar \Omega }{2}\cos[k_{L}(x-\Delta L\sin\omega t)]\left[1+\frac{(\omega_{0}-\omega_{L}{)}^{2}}{2\Omega^{2}\cos^{2}[k_{L}(x-\Delta L\sin\omega t)]}\right]\;+O\left({\left(\frac{\omega_{0}-\omega_{L}}{\Omega }\right)}^{3}\right).$$

These provide the two PESs on which the two-level system evolves, connected by tunnelling. This can be seen by the fact that the intersection of the two curves occurs when $\Omega_{eff}$ is zero, leading to


(2.16) $$x=\Delta L\;\sin\omega t+\frac{\pi }{2k_{L}}+i\log\left(\sqrt{1-\frac{\delta_{L}^{2}}{2\Omega^{2}}}-\frac{\delta_{L}}{\sqrt{2}\Omega }\right)\;≃\Delta L\;\sin\omega t+\frac{\pi }{2k_{L}}-i\frac{\sqrt{2}\delta_{L}}{2\Omega },$$

for small detuning. The binding part of the potential in equation (2.15) supports eigenvalues. However, since the Hamiltonian is periodic in time, the eigenvalues are quasi-energies. Owing to the imaginary part, these are more precisely ‘quasi-energy resonances’.

#### 2.1.2. Large detuning

We consider the case where we have the RWA and adiabatic approximation but $\delta_{L}\gg \Omega$ . Then, we have the Hamiltonian,


(2.17) $$H_{ad,l}^{RWA}=\left(\begin{array}{cc} p^{2}/2m+\hbar \Omega_{eff} & 0\\ 0 & p^{2}/2m-\hbar \Omega_{eff} \end{array}\right).$$

This can be decomposed into two Hamiltonians


(2.18) $$H_{ad,l}^{RWA,\pm }=\frac{p^{2}}{2m}\pm \frac{\hbar \delta_{L}}{2}\left[1+\frac{\Omega^{2}}{2\delta_{L}^{2}}\cos^{2}[k_{L}(x-\Delta L\sin\omega t)]\right]+O\left({\left(\frac{\Omega }{\omega_{0}-\omega_{L}}\right)}^{3}\right).$$

The potential energy curves intersect when


(2.19) $$x(t)=\left(n+\frac{1}{2}\right)\frac{\pi }{k_{L}}+\Delta L\;\sin\omega t.$$

Here, the intersection points are real where the real part is the same as for small detuning. The potential energy curves support sharp quasi-energies.

## 3. Exact solution

We now return to equation (1.6) and lift all the approximations considered in the last section. The Hamiltonian is written as


(3.1) $$H_{rot}=\frac{p^{2}}{2m}I+\left(\begin{array}{cc} a & b\\ b^{*} & -a \end{array}\right)\equiv \frac{p^{2}}{2m}I+M,$$

where $a=\hbar (\omega_{0}-\omega_{L})/2,\;b=b_{1}+ib_{2}$ , with


(3.2) $$b_{1}=\frac{\hbar \Omega }{2}\cos[k_{L}(x-\Delta L\sin\omega t)](1+\cos2\omega_{L}t),\;b_{2}=\frac{\hbar \Omega }{2}\cos[k_{L}(x-\Delta L\sin\omega t)]\sin2\omega_{L}t.$$

The matrix denoted by M in equation (3.1) can be diagonalized by matrix S to get the diagonal matrix, J . These matrices are


(3.3) $$S=\left(\begin{array}{cc} \frac{\left(a-\sqrt{a^{2}+b_{1}^{2}+b_{2}^{2}})(b_{1}+ib_{2}\right)}{b_{1}^{2}+b_{2}^{2}} & \frac{\left(a+\sqrt{a^{2}+b_{1}^{2}+b_{2}^{2}})(b_{1}+ib_{2}\right)}{b_{1}^{2}+b_{2}^{2}}\\ 1 & 1 \end{array}\right)$$

and


(3.4) $$J=\left(\begin{array}{cc} -\sqrt{a^{2}+b_{1}^{2}+b_{2}^{2}} & 0\\ 0 & \sqrt{a^{2}+b_{1}^{2}+b_{2}^{2}} \end{array}\right).$$

We define $\psi_{1}=S^{-1}\psi_{rot}$ with $i\hbar \partial \psi_{rot}/\partial t=H\psi_{rot}$ . The equation for the time evolution of $\psi_{1}$ is


(3.5) $$i\hbar \frac{\partial \psi_{1}}{\partial t}=-iS^{-1}\frac{\partial S}{\partial t}\psi_{1}+S^{-1}\frac{p^{2}}{2m}IS\psi_{1}+J\psi_{1}.$$

Now, $S^{-1}p^{2}S=(S^{-1}pS{)}^{2}=(p-i\hbar S^{-1}\partial S/\partial x{)}^{2}$ . Here, we again have a vector potential which is an artificial gauge field.

The Hamiltonian is thus written as an expansion [64,68]


(3.6) $$H=H_{0}+\hbar H_{1}+\hbar^{2}H_{2}$$

with *H*_0_ has a simple form


(3.7) $$H_{0}=\frac{p^{2}}{2m}\text{I}+\left(\begin{array}{cc} -\sqrt{a^{2}+b_{1}^{2}+b_{2}^{2}} & 0\\ 0 & \sqrt{a^{2}+b_{1}^{2}+b_{2}^{2}} \end{array}\right).$$

Writing $\psi_{1}=(\psi_{1}^{\left(+\right)}\;\;\;\psi_{1}^{\left(-\right)}{)}^{T}$ with the superscript T denoting the transpose, we have written the state with two components. The classical Hamiltonians corresponding to the states, $\psi_{1}^{\left(\pm \right)}$ are


(3.8) $$H_{0}^{\left(\pm \right)}=\frac{p^{2}}{2m}\pm \frac{\hbar (\omega_{0}-\omega_{L})}{2}{\left(1+\frac{4\Omega^{2}}{(\omega_{0}-\omega_{L}{)}^{2}}\cos^{2}[k_{L}(x-\Delta L\sin\omega t)]\cos^{2}\omega_{L}t\right)}^{1/2}.$$

Usually, $\psi_{1}^{\left(+\right)}$ is subjected to a binding potential and $\psi_{1}^{\left(-\right)}$ is evolving on a scattering potential. There are two PESs, $\pm \sqrt{a^{2}+b_{1}^{2}+b_{2}^{2}}$ on which the full two-component wavefunction, $\psi_{1}$ , evolves. The PESs meet at the solution of


(3.9) $$a^{2}+b_{1}^{2}+b_{2}^{2}=0.$$

The solution is


(3.10) $$x=\Delta L\sin\omega t+\frac{1}{k_{L}}\cos^{-1}\left[\pm i\;\frac{\left(\omega_{0}-\omega_{L}\right)}{2\Omega }\sec(\omega_{L}t)\right]\;=\Delta L\sin\omega t+\frac{\pi }{2k_{L}}+i\;\frac{1}{k_{L}}\log\left[1\mp \frac{\delta_{L}}{2\Omega }\sec(\omega_{L}t)+\frac{\delta_{L}^{2}}{8\Omega^{2}}\sec^{2}(\omega_{L}t)\right].$$

For small detuning ( $\delta_{L}\ll \Omega$ ), the potential curves intersect at


(3.11) $$x=\Delta L\sin\omega t+\frac{\pi }{2k_{L}}\mp i\;\frac{\delta_{L}}{2\Omega }\sec(\omega_{L}t)\pm i\;\frac{\delta_{L}^{3}}{48\Omega^{3}}\sec^{3}(\omega_{L}t).$$

The complex value of crossing the PESs implies the tunnelling of atoms. The tunnelling across these surfaces where the underlying dynamics is nonlinear has some very interesting related phenomena like resonance-assisted tunnelling [71], which have been recently experimentally realized [72].

**Figure 1.**
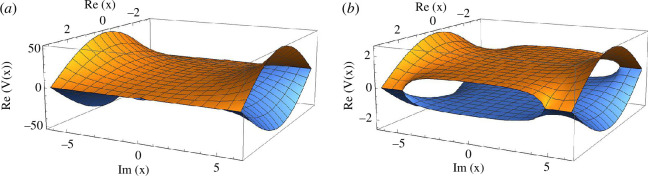
PES at (*a*) large detuning ( $\delta_{L}\gg \Omega$ ) and (*b*) small detuning ( $\delta_{L}\ll \Omega$ ). At large detuning, the gap shrinks allowing a larger region for space for crossing the PES.

Figure 1*a*,*b* shows these crossings along the complex position plane. We note that the crossing gap at the null imaginary position plane vanishes as one reaches closer to the resonance (at small detuning) and remains wide open at large detuning.

In equation (3.8), for large detuning, $\Omega^{2}/(\omega_{0}-\omega_{L}{)}^{2}\ll 1$ , a Taylor expansion immediately yields


(3.12) $$H_{0,l}^{\left(\pm \right)}=\frac{p^{2}}{2m}\pm \frac{\hbar (\omega_{0}-\omega_{L})}{2}\left(1+\frac{2\Omega^{2}}{(\omega_{0}-\omega_{L}{)}^{2}}\cos^{2}[k_{L}(x-\Delta L\sin\omega t)]\cos^{2}\omega_{L}t\right).$$

**Figure 2.**
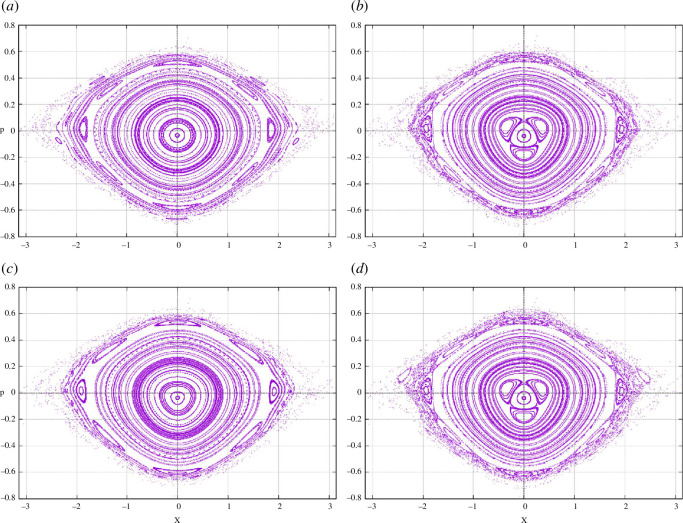
Comparison of Poincaré sections for Hamiltonians under different approximations for the case of large detuning for the same set of parameters used in figure 3. (*a*) Shows the unapproximated case corresponding to the exact solution. (*b*) Shows the application of binomial approximation to the exact solution. *(c*) Corresponds to the RWA + Adiabatic approximation and (*d*) corresponds to the RWA + Adiabatic + Binomial approximation. Initial conditions and number of evolution steps are kept the same for all cases here.

Among the two Hamiltonians, $H_{0,l}^{\left(-\right)}$ is binding; it can be seen that the second term in the Taylor expansion of $\cos[k_{L}(x-\Delta L\sin\omega t)]$ along with an overall negative sign will make this roughly parabolic for small arguments, at least. For the same reason, $H_{0}^{\left(+\right)}$ is a scattering potential. The differences in Poincaré sections, obtained by slicing phase space evolution in the time intervals of the modulation period T=2π/ω for various cases can be seen in figure 2*a*–*d* that are evaluated using numerical simulations (using Runge–Kutta order 4) for the classical equation of motions for Hamiltonians obtained from equations (3.8), (3.12), (2.17), and (2.18), respectively. We found that the three-island ring which is present in both unapproximated case and RWA + Adiabatic case vanishes if we make a binomial approximation implying that the origin of this resonance is purely arising because of higher order terms of equations (3.12) and (2.15). We also note that the chaos is more apparent in the binomial case but less severe in all other cases.

**Figure 3.**
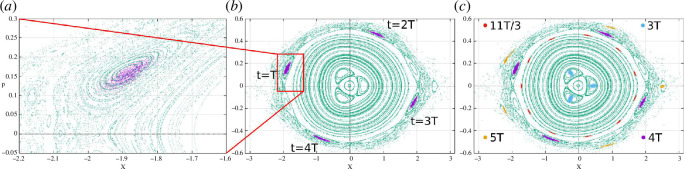
Poincaré sections taken in steps of the modulation period using the same parameter as in [1]. (*a*) 1000 ultracold atoms (purple dots) are loaded in one of the islands of stability in the Poincaré section taken in steps of the driving period T. (*b*) Stroboscopic evolution of the ultracold atoms reveals that they evolve with period 4T. (*c*) Similarly, loading on different islands of stability shows the existence of 3T, 11T/3, 4T and 5T periods predominantly.

We now study the classical mechanics of these Hamiltonians.

## 4. Quasi-integrability

In this section, we study the classical dynamics of the Hamiltonians obtained above under different approximations.

We begin with the exact Hamiltonian, namely, equation (3.6) and consider only $H_{0}^{\left(-\right)}$ in equation (3.8). We make the following transformations to convert it to a dimensionless form almost similar to [63]:


(4.1) $$t\to \frac{t}{\omega }\;,\;x\to \frac{x}{2k_{L}}\;,\;p\to \frac{M\omega p}{2k_{L}}\;,\;H_{0}^{-}\to \frac{M\omega^{2}H_{0}^{-}}{4K_{L}^{2}}\;\lambda =2k_{L}\Delta L\;,\;\gamma =\frac{\omega_{L}}{\omega }\;,\;\eta ={\left(\frac{\Omega }{\delta_{L}}\right)}^{2}\;,\;K=\frac{\hbar k_{L}^{2}\Omega^{2}}{2M\omega^{2}\delta_{L}},$$

where η is the strength of Rabi resonance and $\delta_{L}=\omega_{0}-\omega_{L}$ is the detuning of laser. The simplified Hamiltonian yields


(4.2) $$H_{0}^{-}=\frac{p^{2}}{2}-\frac{4K}{\eta }{\left[1+2\eta (1+\cos(x-\lambda \sin t))\cos^{2}\gamma t\right]}^{\frac{1}{2}}.$$

Now, using the same transformations (equation (4.1)), we write the Hamiltonians for large detuning, neglecting the constant terms,


(4.3) $$H_{0,l}^{-}≃\frac{p^{2}}{2}-4K\cos(x-\lambda \sin t)\cos^{2}\gamma t,$$

(4.4) $$H_{ad,l}^{RWA,-}≃\frac{p^{2}}{2}-K\cos(x-\lambda \sin t).$$

This clearly implies a drastic change in the equation if γ≫1 , thus even if we use $\langle \cos^{2}\gamma t\rangle =1/2$ , the second term contributes to double compared with the contribution coming from the usual case with adiabatic and RWA.

In order to understand the underlying phase space structure, we initialize 1000 ultracold atoms (purple dots) in one of the islands in the Poincarè section taken in steps of modulation time period T as shown in figure 3 *a* and look at its stroboscopic evolution in multiples of the modulation time period. We found that after each modulation period, atoms move from one island to another lying around the same larger elliptic-like orbit (figure 3 *b*). Similarly, we found that the number of islands is equal to (or twice if n is even) the number of modulation periods n for the marked islands in figure 3 *c*. In other words, these islands satisfy $T_{orbit}=nT$ or $\Omega_{orbit}/\omega =1/n$ .

To study the origin of these patterns in resonance structures, we write the dimensionless Hamiltonian equation (4.4) in action-angle variables. Let us write one of the RWA Hamiltonians as a perturbed harmonic oscillator


(4.5) $$H_{0,l}^{RWA,-}=\frac{p^{2}}{2}+\frac{Kx^{2}}{2}-\left(K\cos(x-\lambda \sin t)+\frac{Kx^{2}}{2}\right),$$

(4.6) $$=H_{h.o.}+\epsilon \Delta H,$$

where ϵ is introduced for book keeping (eventually, we shall put ϵ=1 ). Using the oscillator action-angle variables, (J,θ) , with $x=\sqrt{\frac{J}{\pi \Omega }}\sin(\theta )$ and $p=\sqrt{\frac{J\Omega }{\pi }}\cos(\theta )$ with $K=\Omega^{2}$ , the Hamiltonians are


(4.7) $$H_{h.o.}=\frac{\Omega J}{2\pi },$$

(4.8) $$\Delta H=-\Omega^{2}\cos\left(\sqrt{\frac{J}{\pi \Omega }}\sin\theta -\lambda \sin t\right)-\frac{J\Omega }{2\pi }\sin^{2}\theta .$$

We use the classical time-dependent perturbation theory [59] to calculate the associated action of this Hamiltonian up to first order in perturbation. For this, we transform the action variables in a way that the new Hamiltonian $\bar{H}$ is only a function of the new action variable $\bar{J}$ alone. We obtain


(4.9) $$\left\langle \Delta H\rangle =\frac{1}{2\pi }\int_{\;\;0}^{2\pi }dt\frac{1}{2\pi }\int_{\;\;0}^{2\pi }d\theta \Delta H(J,\theta ,t\right)\;=-\Omega^{2}J_{0}\left(\sqrt{\frac{\bar{J}}{\Omega \pi }}\right)J_{0}(\lambda )-\frac{\bar{J}\Omega }{4\pi }$$

(4.10) $$\bar{H}(\bar{J})=\frac{\Omega \bar{J}}{2\pi }-\epsilon \Omega^{2}J_{0}\left(\sqrt{\frac{\bar{J}}{\Omega \pi }}\right)J_{0}(\lambda )-\epsilon \frac{\bar{J}\Omega }{4\pi }$$

where $J_{0}(.)$ is the cylindrical Bessel function of order zero. The new frequency is


(4.11) $$\Omega^{\prime }(\bar{J})=2\pi \frac{\partial \bar{H}}{\partial \bar{J}}=\Omega (1-\epsilon /2)-2\epsilon \pi \Omega^{2}J_{0}^{\prime }\left(\sqrt{\frac{\bar{J}}{\Omega \pi }}\right)J_{0}(\lambda )$$

where prime on the Bessel function denotes a derivative with respect to its argument.

We subtract this ϵ⟨ΔH⟩ from ϵΔH to obtain the oscillating part ϵ{ΔH} . For calculating the integral, we expand the potential term using Jacobi–Anger expansion [73] $e^{iz\sin\theta }=\sum_{n=-\infty }^{+\infty }J_{n}(z)e^{in\theta }$ :


(4.12) $$\{\Delta H\}=-\sum_{n,m=-\infty }^{\infty }\Omega^{2}J_{n}\left(\sqrt{\frac{\bar{J}}{\Omega \pi }}\right)J_{m}(\lambda )\cos(n\bar{\theta }-mt)+\frac{\bar{J}\Omega }{4\pi }\cos2\bar{\theta }$$

(4.13) $$\equiv \sum_{n,m=-\infty }^{\infty }\Delta H_{n,m}(\bar{J},\bar{\theta },t)+\frac{\bar{J}\Omega }{4\pi }\cos2\bar{\theta },$$

where both n,m are non-zero. The change in action ϵΔS can be calculated as


4.14) $$\epsilon \Delta S=-\int^{t}dt\epsilon \{\Delta H\}$$

(4.15) $$=\sum_{n,m=-\infty }^{\infty }\epsilon \Delta S_{n,m}(\bar{J},\bar{\theta },t)+\frac{\epsilon \bar{J}\Omega }{8\pi \bar{\Omega }(\bar{J})}\sin2\bar{\theta },$$

where


(4.16) $$\epsilon \Delta S_{n,m}=\frac{-\epsilon \Omega^{2}}{n\bar{\Omega }(\bar{J})-m}J_{n}\left(\sqrt{\frac{\bar{J}}{\Omega \pi }}\right)J_{m}(\lambda )\sin(n\bar{\theta }-mt).$$

Consequent to the above,


(4.17) $$\bar{J}=J-\epsilon \frac{\partial \Delta S}{\partial \theta }(J,\theta ,t)\;;\;\bar{\theta }=\theta +\epsilon \frac{\partial \Delta S}{\partial J}(J,\theta ,t).$$

The new action-angle variables can be calculated up to first order as


(4.18) $$\bar{J}=J+\epsilon \frac{n\Omega^{2}}{n\bar{\Omega }(J)-m}J_{n}\left(\sqrt{\frac{J}{\Omega \pi }}\right)J_{m}(\lambda )\cos(n\theta -mt)-\epsilon \frac{J\Omega }{4\pi }\cos2\theta ,$$

(4.19) $$\bar{\theta }=\theta +\epsilon \frac{-\Omega^{2}}{n\bar{\Omega }(J)-m}J_{n}^{\prime }\left(\sqrt{\frac{J}{\Omega \pi }}\right)J_{m}(\lambda )\sin(n\theta -mt)+\frac{\epsilon \Omega }{8\pi \bar{\Omega }(\bar{J})}\sin2\theta .$$

**Figure 4.**
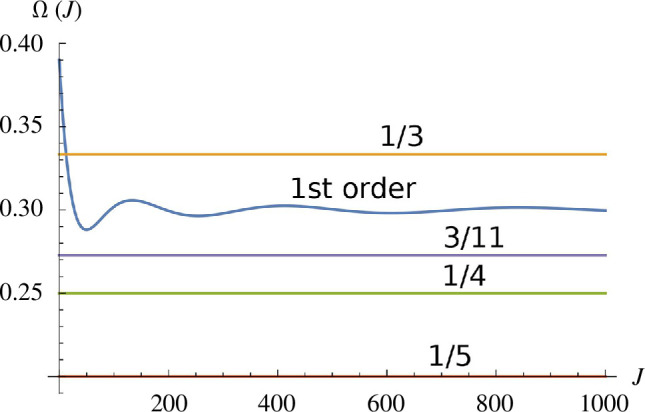
First order correction in Ω(J) . Only those resonances whose frequency ratio Ω(J):ω ( ω =1 here) intersect with Ω(J) are allowed.

Thus, we have obtained the action with resonant denominators which leads to the resonant condition


(4.20) $$n\bar{\Omega }(\bar{J})=m\omega ,$$

where ω is the modulation frequency and $\bar{\Omega }(\bar{J})$ is the frequency of the orbit; ω is obtained when we substitute the actual time, t , in place of dimensionless time from equation (4.1). This explains the observed pattern in figure 3: the orbital periods are the integral multiples of the modulation period at the resonance. The strength of (n,m)th resonance is determined by the product of two Bessel functions $J_{n}(\sqrt{J/\Omega \pi })$ and $J_{m}(\lambda )$ . Using the first-order correction in the frequency Ω(J) , we plot it as a function of J in figure 4. We see that only the 1:3 resonance is allowed under first-order correction. This means that all other resonances in figure 3 must originate from the higher-order perturbation terms in correction for $\bar{\Omega }$ and $\bar{J}$ . That explains the dominance of primary islands in (n,m)=(3,1) resonance and the presence of secondary islands in other resonances.

For the expression without binomial approximation equation (4.4), where in figure 2, we saw (3,1) resonance to be dominantly present, but without binomial approximation (equation (2.17)), this resonance is suppressed and does not appear. This can lead to significant corrections for both quantum and classical equations despite being in a large detuning limit. Similarly, very high-ordered resonances are enhanced by binomial approximation as the chaotic regime can be seen enhanced around the edges for this case.

## 5. Dynamical localization

Let us imagine that we prepare the initial state of the atoms as a localized wavepacket. As the system evolves, the wavepacket spreads. The wavefunction of the two-state system is shown to evolve, in all versions of description, on a pair of PESs. The form of these potentials readily supports the bounded dynamics of one of the potentials. The complex intersection points provide paths for tunnelling. The succession of these two dynamical features leads to the localization of the wavepacket. The physics of this is nothing but the well-known argument by Mott & Twose [74] and Anderson [54], adapted in recent times in quantum chaos [55,56].

## 6. Conclusions

When a collection of atoms is subjected to phase modulation, the quantum and classical dynamics are dictated by several frequencies. The quantum dynamics of the two-level systems has been studied in the past where the main result was the observation of dynamical localization by Raizen’s group [1]. The theoretical analysis of the system has been carried out under various approximations and discussed at a didactic level [63]. Here, we perform the analysis of this system by using successive unitary transformations on the off-diagonal part of the Hamiltonian in the process of diagonalization, as explained in §3. We have worked with the transformed Hamiltonian which is diagonal to O(1). Our analysis, following earlier works in chemical physics and nuclear physics, explained in detail in a comprehensive review [64], shows that the dynamics of the two-state system take place on coupled PESs. The connection between the two surfaces occurs via tunnelling and the underlying classical dynamics is shown to be quasi-integrable of the KAM type. This is brought out by the Poincaré surfaces of sections where we note the presence of elliptic and hyperbolic points, typically paraphrased as dynamics occurs in the mixed-phase space with ‘stable islands in the stochastic sea’. A detailed understanding of dynamics is interesting and illuminating.

Let us comment about the usage of the term ‘exact’. Upon diagonalization of the potential matrix, there appears a ‘vector potential’ in the kinetic energy term, leading to terms in orders of Planck’s constant. The transformed Hamiltonian is $\left[(p-\hbar A{)}^{2}/2+v\right]$ . The important point is that $(p^{2}/2m+v)I$ is diagonal whereas the other terms of orders ℏ and $\hbar^{2}$ are not diagonal. Our objective is to diagonalize the Hamiltonian matrix. At this step, the diagonalization is up to O(1). This process can be repeated by diagonalizing the off-diagonal terms in **A.p** and **A.A** by successive unitary transformations. In principle, this process can be repeated ad infinitum, leading to complete diagonalization.

The approximated analysis has certain appeal insofar as tunnelling between islands is seen clearly. However, to establish the existence of islands and tunnelling, we show that the onset of islands of stability can be seen from the first-order perturbation theory.

As explained above, the analysis reveals a vector potential that is related to an artificial gauge field. We believe that knowing the form of this could be useful for experiments with cold atoms and in developing the fields of Hamiltonian engineering, quantum sensing and quantum interference. We have not developed these aspects here.

As referred to in the Introduction, our results add to the discussion of integrability in matrix models for atomic systems, in particular to the work on the quantum Rabi model [61]. In the future, by adding nonlinear terms to incorporate interactions that allow the control of atomic states, these works could be useful for critical quantum metrology [75]. The control of states of multi-qubit systems [76] and their protection [77] belongs to the present theme in a rather compelling manner.

## Data Availability

This article has no additional data.

## Footnotes

We can make a rough estimate of relevant classical action by considering the product of momentum of the trapped sodium atom and the size of the trap. With the values corresponding to the experiment [1]. The momentum corresponding to 17 µK is approximately 4.23 × 10^−27^ kg m s^−1^. In a spherical trap of radius, 300 µm, the ratio of ~ to the action is estimated to be about 10^− 5^. If the atom remains in a smaller region, this ratio would still be at least 10^− 4^ or so. A semiclassical expansion is certainly justified.

By classical correspondence, we assume the validity of Ehrenfest’s theorem.
